# Genomic Sequencing and Analysis of *Sucra jujuba* Nucleopolyhedrovirus

**DOI:** 10.1371/journal.pone.0110023

**Published:** 2014-10-17

**Authors:** Xiaoping Liu, Feifei Yin, Zheng Zhu, Dianhai Hou, Jun Wang, Lei Zhang, Manli Wang, Hualin Wang, Zhihong Hu, Fei Deng

**Affiliations:** State Key Laboratory of Virology, Virus Resource and Bioinformatics Center, Wuhan Institute of Virology, Chinese Academy of Sciences, Wuhan, Hubei, China; Ecole des Mines d'Alès, France

## Abstract

The complete nucleotide sequence of *Sucra jujuba* nucleopolyhedrovirus (SujuNPV) was determined by 454 pyrosequencing. The SujuNPV genome was 135,952 bp in length with an A+T content of 61.34%. It contained 131 putative open reading frames (ORFs) covering 87.9% of the genome. Among these ORFs, 37 were conserved in all baculovirus genomes that have been completely sequenced, 24 were conserved in lepidopteran baculoviruses, 65 were found in other baculoviruses, and 5 were unique to the SujuNPV genome. Seven homologous regions (*hr*s) were identified in the SujuNPV genome. SujuNPV contained several genes that were duplicated or copied multiple times: two copies of helicase, DNA binding protein gene (*dbp*), *p26* and *cg30,* three copies of the inhibitor of the apoptosis gene (*iap*), and four copies of the baculovirus repeated ORF (*bro*). Phylogenetic analysis suggested that SujuNPV belongs to a subclade of group II alphabaculovirus, which differs from other baculoviruses in that all nine members of this subclade contain a second copy of *dbp*.

## Introduction

Baculoviruses are rod-shaped, insect-specific viruses with double-stranded, circular DNA 80–180 kb genomes [1]. Baculoviruses have been widely used as bio-pesticides to control insect pests in agriculture and forestry [2], as vectors for protein expression, and as potential vectors for gene therapy [3], [4]. The family Baculoviridae used to be grouped into two genera: Nucleopolyhedroviruses (NPVs) and Granuloviruses (GVs), dependent upon differing morphologies of occlusion bodies (OBs) [5]. More recently, a new classification has subdivided the Baculoviridae into four genera, based on phylogeny and host specificities: Alphabaculovirus (lepidopteran-specific NPVs), Betabaculovirus (lepidopteran-specific GVs), Gammabaculovirus (hymenopteran-specific NPVs) and Deltabaculovirus (dipteran-specific NPVs) [6]. Alphavaculoviruses can be further gathered into group I and group II based on phylogenetic analyses, The NPVs are also characterized as single nucleocapsid NPVs (SNPVs) and multiple-nucleocapsid NPVs (MNPVs) according to the number of nucleocapsids per virion. To date, 62 baculovirus reference genomes are available in the National Centre for Biotechnology Information (NCBI) database; 42 of them are alphabaculoviruses, 15 betabaculoviruses, three gammabaculoviruse*s,* one deltabaculovirus and one unclassified baculovirus.

*Sucra jujuba* Chu (Lepidopteral: Geometridae) is an important pest of jujube, and it is widespread in the jujube-growing regions of China. The larvae feed on the young leaves and buds of jujube, apple, pear and mulberry. In 2009, 1250 square hectometers of mulberry became infested with *Sucra jujuba* Chu in China [7]. *Sucra jujuba* NPV (SujuNPV) is a SNPV, which was first isolated from naturally diseased *Sucra jujuba* larvae in the early 1980s [8]. The virus is highly infectious to *Sucra jujuba* with an LC_50_ of 3.5×10^5^ PIBs/mL in the third instar larvae [9]. It appears to be specific to *Sucra jujuba* as bioassay studies showed that it did not infect *Antheraea pernyi, Arge captiva, Bombyx mori, Culcula panterinaria*, *Euproctis flava*, *Leucoma salicis, Lymantria dispar, Macaria elongaria, Phthonandria atrilineata*, *Plusia agnate* or *Semiothisa cinerari*a [8], [10], [11].

In the present study, the genome of SujuNPV is completely sequenced and annotated, and compared with those of the other representative baculoviruses. Results indicate that SujuNPV is a novel species belonging to a unique subclade of group II alphabaculoviruses, which contain a second copy of the DNA binding protein gene (*dbp*).

## Materials and Methods

### DNA extraction of the viral genome

The SujuNPV were purified from the dead *Sucra jujube* preserved in “Chinese general virus collection center” (CGVCC) with collection Number IVCAS 1.0048, which was originally isolated from Shandong Province, China, in 1983 [12]. The ODVs were purified as previously reported [13]. To extract DNA, the ODVs were incubated with four times volume 1 M DAS (5 M Nacl, 5 M NaCO_3_ and 0.5 M EDTA (pH8), mixed in the ratio of 3∶3∶0.6) at 37°C for 30 min. Then, the same volume of 1 M Tris (pH 7.4) was added followed by centrifugation at 10,000 rpm (5 min) to obtain the viral DNA.

### Sequencing and sequence analysis of the SujuNPV genome

The SujuNPV genome sequence was determined by 454 pyrosequencing. A total of 92,684 reads were obtained and assembled into 10 contigs using GS De Novo Assembler software, covering 97.8% of the whole genome with a sequencing depth of 225x. The remaining gaps were filled using PCR and Sanger sequencing.

Briefly, the genome was broken randomly into small fragments of about 600–900 bp by nebulization and adapters were added to construct a genomic library. Subsequently, the library was amplified by emPCR before sequencing. The SujuNPV genome was assembled using a GS De Novo Assembler providing 454 programs. Additional verifications were performed for gaps and ambiguous sequences using sequence-specific primers. The hypothetical ORFs of the SujuNPV genome were predicted by fgenesV0 (http://www.softberry.com/berry.phtml) [14], adopting the criteria of a size of at least 50 aa with a minimal overlap with other ORFs. Predicted aa sequences were compared with homologues of typical baculoviruses of the four genera, including AcMNPV (NC_001623), HearNPV-G4 (NC_002654), CpGV (NC_002816), NeleNPV (NC_005906) and CuniNPV (NC_003084), and similarities were obtained by DNAStar software with default parameters.

Gene parity plots were generated in order to analyze the gene order of SujuNPV relative to three other closely related baculoviruses (ApciNPV, EcobNPV and OrleNPV) and the five representative viruses mentioned above.

Consensus promoter motifs were searched for in the upstream 150 bp region from the start codon of each ORF based on the characterization of baculovirus' promoters, that’s a TATA box linked with a CAKT motif 20–40 bp downstream and a DTAAG box.

### Phylogenetic analysis

Phylogenetic analysis of baculoviruses was performed using the concatenated aa sequence of 37 core genes [15] from 62 baculovirus reference genomes (http://www.ncbi.nlm.nih.gov/genomes/GenomesGroup.cgi?taxid=10442, data update until Jan.5^th^, 2014). The sequences were aligned by ClustalW with default parameters of MEGA5. And the maximum likelihood (ML) phylogenetic tree was reconstructed according to the previous report [16] with 1000 bootstrap values. The phylogenetic trees of *dbp*, *helicase* and *p26* were constructed based on the same parameters.

### Prediction of secondary structure

The secondary structures of DNA sequences were predicted by the Mfold Web Server using default parameters [17].

## Results and Discussion

### Characteristics of the SujuNPV genome sequence

**Figure 1:**
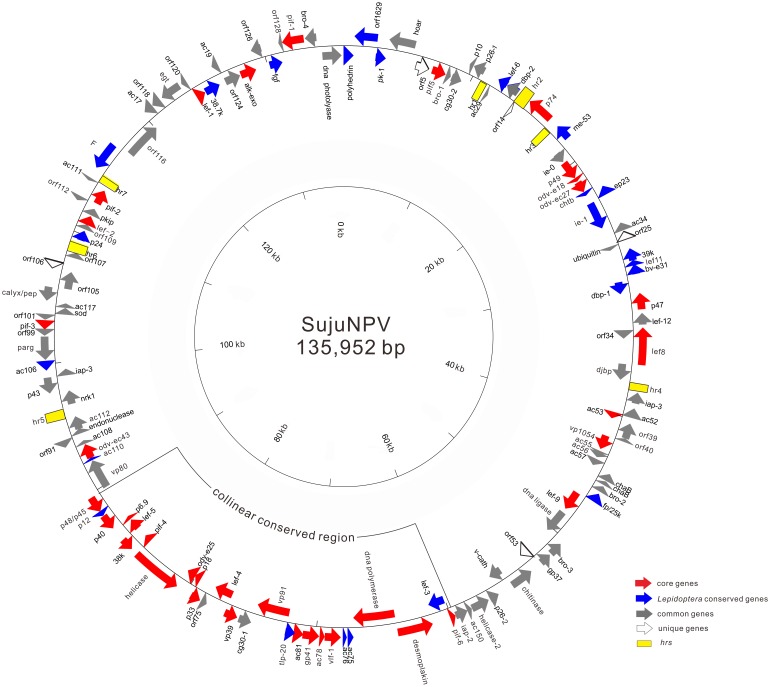
Circular map of the SujuNPV genome. The arrows inside or outside the circle indicate the orientation of putative ORFs. Arrows, red represent the core genes, blue represent *Lepidoptera* baculovirus conserved genes, grey represent genes common to baculoviruses, open are genes unique to SujuNPV, and yellow rectangles indicate *hrs*. The collinear region conserved in *Lepidoptera* baculoviruses is also shown.

The full SujuNPV genome [GeneBank: KJ676450] was 135,952 bp in length with an A+T content of 61.34%. Following convention, the adenine coding for the start methionine of the *polyhedrin* gene (*ph*) was chosen as the zero point of the SujuNPV genome and *ph* was designated as the first ORF. Overall, 131 putative ORFs were detected in the SujuNPV genome with the criteria of a length of at least 50 amino acids (aas) and a minimal overlap with adjacent ORFs. The total ORFs covered 89.2% of the whole genome, distributed with 60 ORFs in a forward orientation and 71 ORFs in a reverse orientation. In addition, seven homologous regions (*hr*s) were identified in SujuNPV (Fig. 1).

BLAST comparisons of the 131 protein sequences of the SujuNPV, deduced from the homologous sequences of other baculoviruses, revealed that SujuNPV has 37 core genes (shown in red in Fig. 1) and 24 other genes conserved in lepidopteran baculoviruses (shown in blue in Fig. 1). It also contains 65 additional genes commonly found in various baculoviruses (shown in grey in Fig. 1) and five unique genes (shown as open arrows in Fig. 1). Consensus promoter motifs were searched for in the upstream 150 bp region of the start codon of each ORF. Amongst all 131 ORFs identified in the SujuNPV genome, 24 ORFs possessed the early promoter motif (a TATA box linked with a CAKT motif 20–40 bp downstream), whereas 61 ORFs had the late promoter motif DTAAG and 10 ORFs contained both the early and late promoter motifs (Table 1). No obvious baculoviral promoter motifs were detected for the remaining 36 ORFs.

**Table 1:** SujuNPV Genome Annotation.

ORF	name	motif	start	end	length (aa)	str.	ORF position					amino acid identity (%)				
							AcMNPV	HearNPV	CpGV	NeleNPV	CuniNPV	AcMNPV	HearNPV	CpGV	NeleNPV	CuniNPV
1	*polyhedrin*	E	1	741	246	+	8	1	1	1		86.5	88.2	53.7	45.1	
2	*orf1629*	L	774	2474	566	−	9	2	2			14.9	19.6	17.2		
3	*pk-1*		2467	3270	267	+	10	3	3			32.6	44.6	30.3		
4	*hoar*	E	3313	5259	506	−		4					15			
5	*orf5*		5663	6724	353	+										
6	pif-5*	L	6962	8059	365	+	148	15	18	23	102	53.4	53.7	45.1	37.5	19.7
7	*bro-1*	L	8122	8466	114	+		59					23.7			
8	*cg30-2*		8570	9388	272	+	88	77				15.5	12.1			
9	*p10*	L	9436	9696	86	−	137	21	22			27.4	35.7	31		
10	*p26-1*	E,L	9747	10604	285	−	136	22				34.6	43.8			
	*hr1*		10666	11269												
11	*ac29*		11438	11656	72	+	29	23				25.4	40.3			
12	*lef-6*	L	11762	12508	248	−	28	24	80			19.7	26.2	16.8		
13	*dbp-2*		12532	13464	310	−	25	25	81	14		18.1	23.9	14.1	14.8	
	*hr2*		13546	14517												
14	*orf14*		13638	13889	83	+										
15	p74*	L	14521	16479	652	−	138	20	60	47	74	57.8	54.4	39.4	38.9	32.8
	*hr3*		16580	17170												
16	*me-53*	E	17219	18349	376	−	139	16–17	143			16.8	22.6	17.2		
17	*ie-0*	E,L	18698	19495	265	+	141	8				24.5	27.5			
18	p49*	L	19595	21028	477	+	142	9	15	60	30	44.4	55.8	26.3	19.2	6.3
19	odv-e18*	L	21058	21324	88	+	143	10	14	62	31	61.3	51.9	38.1	18.8	7.9
20	odv-ec27*	L	21428	22306	292	+	144	11	97	63	32	45.9	52.1	22.2	21	15.3
21	*chtb*	L	22336	22614	92	+	145	12	9	64		45.5	44.6	31.5	27.2	
22	*ep23*	L	22639	23262	207	−	146	13	8			29.4	25.6	19.3		
23	*ie-1*	E	23325	25439	704	+	147	14	7			24.9	27.2	11.7		
24	*ac34*	L	25586	26134	182	−	34	27				23.6	50			
25	*orf25*	E	26224	26802	192	−										
26	*ubiquitin*	L	26988	27254	88	+	35	28	54			74	72.3	68.2		
27	*39k*		27529	28428	299	−	36	31	57			33.1	35.5	5.8		
28	*lef-11*		28430	28786	118	−	37	32	58			33	30.5	27.1		
29	*bv-e31*	L	28711	29433	240	−	38	33	69			53.7	48.3	39.5		
30	*dbp-1*	E,L	29783	30712	309	+	25	25	81			24.9	34	12.4		
31	p47*		30827	32011	394	−	40	35	68	46	73	53.6	50.8	45.2	25.2	14
32	*lef-12*	E	32272	33114	280	−	41	36				30.9	29.6			
33	lef-8*		33321	36038	905	−	50	38	131	78	26	63	68.9	49.1	30.5	18.3
34	*orf34*		33504	34118	204	+	34	orf34								
35	*djbp*		36078	37316	412	+	51	39				15.4	20.1			
	*hr4*		37323	37964												
36	*iap-1*	L	38038	38844	268	−	27	103	17	11	17	24.3	23.1	24.3	12.7	3.1
37	*ac52*		39221	39895	224	−	52	42				19.5	30.6			
38	*ac53*	L	39855	40328	157	+	53	43	134	77	28	46	52.2	17.3	12.1	8.9
39	*orf39*	L	40332	41459	375	−		44					17.3			
40	*orf40*	L	41473	41709	78	−										
41	*vp1054*	L	41760	42869	369	+	54	47	138	83	8	39.7	47.9	30.1	18.8	18.5
42	*ac55*		43039	43242	67	+	55	48				35.8	56.7			
43	*ac56*	L	43184	43534	116	+	56	49				11.9	32.8			
44	*ac57*	E,L	43717	44208	163	+	57	50				35.4	38.7			
45	*chaB*	E,L	44279	44800	173	−	59	51				37.7	34.4			
46	*chaB*	L	44876	45145	89	−	60	52				41.4	39.8			
47	*bro-2*	L	45281	45697	138	−		60					27.5			
48	*fp/25k*	L	45905	46549	214	−	61	53	118			51.9	56.1	28		
49	*lef-9*		46687	48216	509	+	62	55	117	37	59	66.2	66.6	52.3	34	18.9
50	*dna ligase*		48547	50361	604	+			120					23.5		
51	*bro-3*		50436	51428	330	−	2	105				36	13.3			
52	*gp37*	E	51534	52391	285	−	64	58	13			45.3	55.6	41.4		
53	*orf53*		52471	53133	220	+										
54	*chitinase*	L	53319	55037	572	−	126	41	10			68.8	63.7	57.3		
55	*v-cath*	L	55144	56136	330	+	127	56	11			66.6	47	42.4		
56	*p26-2*	E	56200	56919	239	−	136	22				17.2	15.9			
57	*helicase-2*		57035	58390	451	−			126					27.5		
58	*ac150*	L	58435	58752	105	−	150	12	79			25.3	21.7	25.7		
59	*iap-2*	E	58756	59682	308	−	71	62	17			28.9	35.2	16		
60	*pif-6*		59711	60088	125	−	68	64	114	38	58	35.2	44	24	21.6	16
61	*lef-3*		60087	61340	417	+	67	65	113			17.9	23.7	4.5		
62	*desmoplakin*	L	61434	64043	869	−	66	66	112	21	92	17.6	17.1	12.3	11.7	10.7
63	*dna polymerase*		64042	67248	1068	+	65	67	111	20	91	44.5	51.6	30.9	24.3	16.5
64	*ac75*	L	67287	67679	130	−	75	69	108			20	34.6	10.8		
65	*ac76*	L	67774	68031	85	−	76	70	107			41.7	65.9	35.7		
66	*vlf-1*	L	68185	69354	389	−	77	71	106	42	18	67.5	69.4	28.6	25.1	18.2
67	*ac78*	L	69378	69710	110	−	78	72	105	43	34	33	45.5	13.6	21.3	16.7
68	*gp41*	L	69758	70963	401	−	80	73	104	44	33	43.1	53.4	28.4	26.3	11.5
69	*ac81*		70932	71621	229	−	81	74	103	45	106	48.9	52.4	41.9	33.1	16.1
70	*tlp-20*	L	71515	72237	240	−	82	75	102			26.7	33.8	11.6		
71	*vp91*	L	72206	74731	841	+	83	76	101	82	35	37.6	42.8	21.8	23.4	22.3
72	*cg30*	E	74792	75739	315	−	88	77				19.7	18.7			
73	*vp39*	L	75861	76835	324	−	89	78	96	88	24	36.4	42	22.5	19	14.5
74	*lef-4*		76834	78264	476	+	90	79	95	59	96	45.7	45.1	30.3	25	13.7
75	*orf75*	L	78301	78726	141	−		77					9.9			
76	*p33*	E,L	78822	79580	252	−	92	80	93	16	14	50.4	57.9	35.1	19.4	19
77	*p18*	L	79579	80055	158	+	93	81	92	17	13	52.5	62	31	17.1	4.8
78	*odv-e25*	L	80057	80728	223	+	94	82	91	18	15	39	59.2	48.8	13	11.2
79	*helicase-1*	L	80777	84505	1242	−	95	84	90	58	89	41	46.9	22.5	17.4	11.1
80	*pif-4*		84459	84980	173	+	96	85	89	57	90	50.3	57.8	35.4	26.2	25.4
81	*38k*	L	85007	85927	306	−	98	86	88	56	87	42.8	50.3	39.5	27.2	24.8
82	*lef-5*	L	85811	86662	283	+	99	87	87	55	88	46.8	54.4	38	27.2	7.9
83	*p6.9*		86739	86999	86	+	100	88	86	28	23	7.3	8.1	4.1	8.1	13.8
84	*p40*	E,L	87012	88154	380	−	101	89	85	29	22	40.2	41.2	17.6	13.7	7.6
85	*p12*	L	88194	88553	119	−	102	90	84			21.8	20.2	13.8		
86	*p48/p45*	E,L	88540	89727	395	−	103	91	83	31	55	42.4	47.5	31.9	15.6	6
87	*vp80*		89820	92120	766	+	104	92				12	17.2			
88	*ac110*		92160	92334	57	+	110	93	53			28.6	40.4	25		
89	*odv-ec43*	L	92343	93482	379	+	109	94	55	67	69	48.3	58.4	30.4	15.3	10.3
90	*ac108*	L	93532	93789	85	+	108	95				29.4	41.2			
91	*orf91*		93807	94313	168	−										
92	*endonuclease*	L	94403	94762	119	+	79		65			24		26.6		
93	*ac112*		94746	95765	339	+	112					31				
	*hr5*		95796	96554												
94	*nrk1*		96753	97811	352	+	33		16			26.4		29.6		
95	*p43*	E	97850	99010	386	−	39					18.2				
96	*iap-3*		99009	99479	156	+	27	103	94			23.1	24.4	27.6		
97	*ac106*	E,L	99518	100228	236	−	106	101	52	32		50.8	49.6	25.4	16.5	
98	*parg*	L	100286	101974	562	−		100					16.7			
99	*orf99*		102055	102522	155	−		99					17.8			
100	*pif-3*	L	102512	103135	207	−	115	98	35	66	46	44.1	47.2	35.7	28.5	31.5
101	*orf101*		103207	103581	124	−										
102	*sod*	L	103683	104159	158	+	31	106	59			60.9	62.7	46.2		
103	*ac117*		104202	104513	103	+	117	110				22.1	39.8			
104	*calyx/pep*	L	104559	105578	339	−	131	120	22	50		22.6	35.7	12.1	12.1	
105	*orf105*		105634	106980	448	+										
106	*orf106*		107008	107532	174	−										
107	*orf107*	L	108086	108496	136	+		68					13.2			
	*hr6*		108513	109329												
108	*p24*	L	109521	110312	263	+	129	118	71			37.9	52	24.1		
109	*orf109*	L	110450	110818	122	+										
110	*lef-2*		110826	111515	229	+	6	117	41	54	25	36.2	36.7	18.7	15.4	11.6
111	*pkip*	L	111630	112145	171	+	24	130				13	29.6			
112	*orf112*		112189	112524	111	−										
113	*pif-2*	L	112615	113769	384	+	22	132	48	52	38	60.2	68.1	50.3	44.3	46.4
114	*ac111*		113827	114033	68	−	111	116				55.2	30.9			
	*hr7*		114159	114766												
115	*F*	E,L	114888	116945	685	−	23	133	31		104	14.2	37.8	24.5		15.3
116	*orf116*		117225	120083	952	+		129					24.6			
117	*ac17*		120196	120909	237	−	17	128				16.5	29.1			
118	*orf118*	E,L	120959	121600	213	−		127					21.4			
119	*egt*	E	121812	123353	513	−	15	126	141			45.1	51.7	35.5		
120	*orf120*	L	123560	123916	118	−										
121	*lef-1*		124016	124717	233	+	14	124	74	65	45	35.6	42.1	33.9	28.4	21.5
122	*38.7k*		124783	126009	408	+	13	123	73			22.9	31.9	13.1		
123	*ac19*	L	126065	126508	147	−	19	115				21.3	23.3			
124	*orf124*	L	126507	127745	412	+										
125	*alk-exo*	L	127807	129066	419	+	133	114	125	33	54	36.3	40.3	32.4	24.4	21.5
126	*orf126*	L	129111	129875	254	−										
127	*fgf*		130158	131180	340	+	32	113	123			23.8	19.6	9.1		
128	*orf128*		131201	131440	79	−		112								
129	*pif-1*	L	131447	133045	532	−	119	111	75	76	29	52.1	46.4	33.3	29.1	26
130	*bro-4*		133123	133962	279	−	2					13.6				
131	*dna photolyase*	E	134303	135796	497	+										

ORFs listed are those predicted in the SujuNPV genome and their homologues in the five representative genomes (AcMNPV, HearNPV-G4, CpNPV, NeleNPV and CuniNPV). The start gene is polyhedrin and core genes were marked with*. E and L indicate the Early and Late promoter motifs, respectively. ‘+’ and ‘−’ means the transcription direction; ‘+’ clockwise; ‘−’ anticlockwise.

### Relationship with other baculoviruses

**Figure 2:**
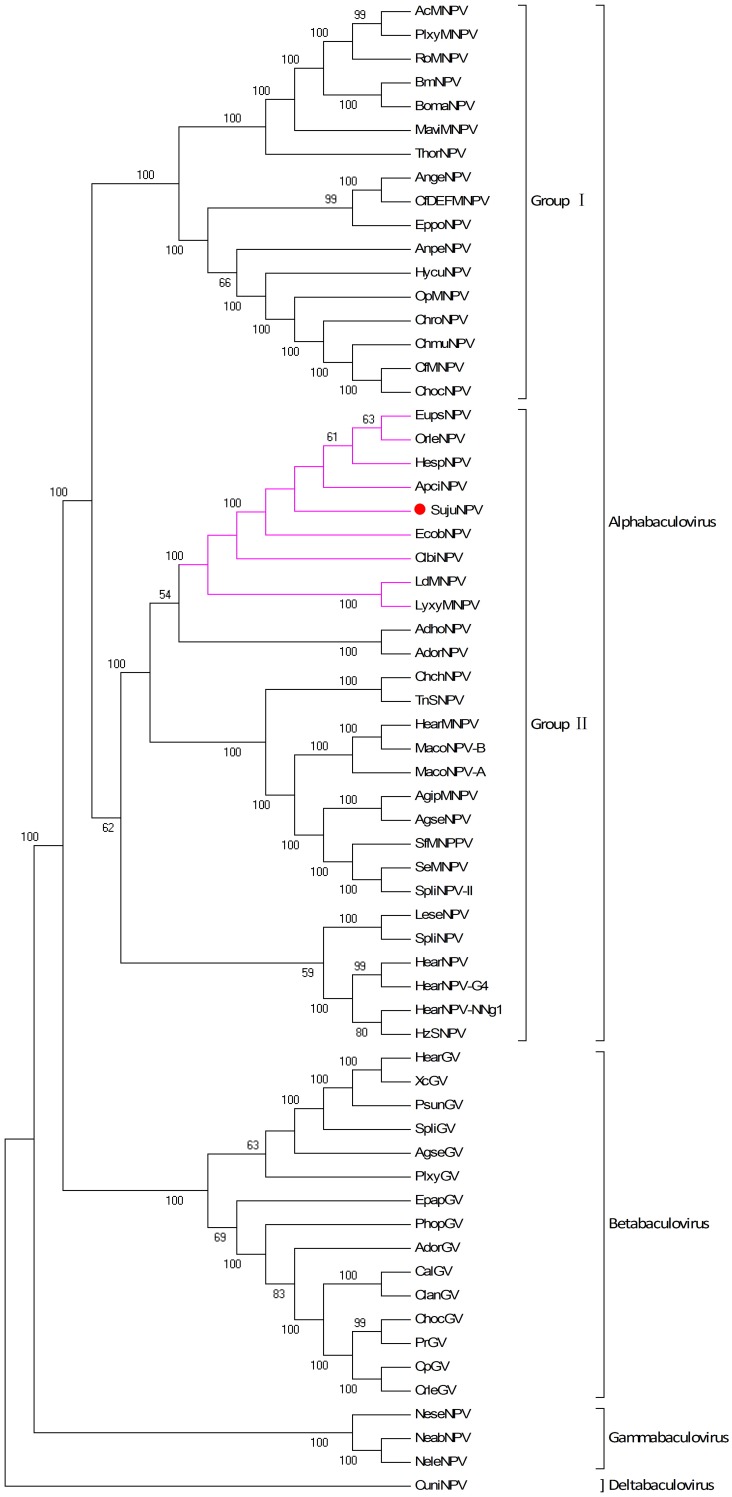
Phylogenic analysis of 62 complete baculovirus genomes. The maximum likelihood (ML) tree was generated based on the concatenated protein sequences of 37 core genes with default parametes and 1000 randoms. The SujuNPV was labeled by a red point and the number on the branch means bootstrap values (only the values over 50 were shown). Pink branches indicate the unique subclade containing a second copy of *dbp*.

Phyogenetic analysis of the 37 core genes of the 62 reference baculoviruses revealed that SujuNPV is a group II alphabaculovirus (Fig. 2). The virus is a novel member of a subclade containing eight other baculoviruses, including *Apocheima cinerarium* NPV (ApciNPV), *Clanis bilineata* NPV (ClbiNPV) [18], *Ectropis obliqua* NPV (EcobNPV) [19], *Euproctis pseudoconspersa* NPV (EupsNPV) [20], *Hemileuca* sp. NPV (HespNPV) [21], *Lymantria dispar* MNPV (LdMNPV) [22], *Lymantria xylina* MNPV (LyxyMNPV) [23] and *Orgyia leucostigma* NPV (OrleNPV) [24].

Five representative baculoviruses were chosen for the comparative study of SujuNPV: *Autographa californica* MNPV (AcMNPV, group I alphabaculovirus) [25], *Helicoverpa armigera* SNPV (HearNPV, group II alphabaculovirus) [26], *Cydia pomonella* GV (CpGV, betabaculovirus), *Neodiprion lecontei* NPV (NeleNPV, gammabaculovirus) and *Culex nigripalpus* NPV (CuniNPV, deltabaculovirus). SujuNPV shared 102 ORFs with AcMNPV, 108 with HearNPV, 78 with CpGV, 43 with NeleNPV, and 39 with CuniNPV, with an average amino acid (aa) identity of 36.0%, 39.0%, 28.4%, 23.0% and 16.3%, respectively.

**Figure 3:**
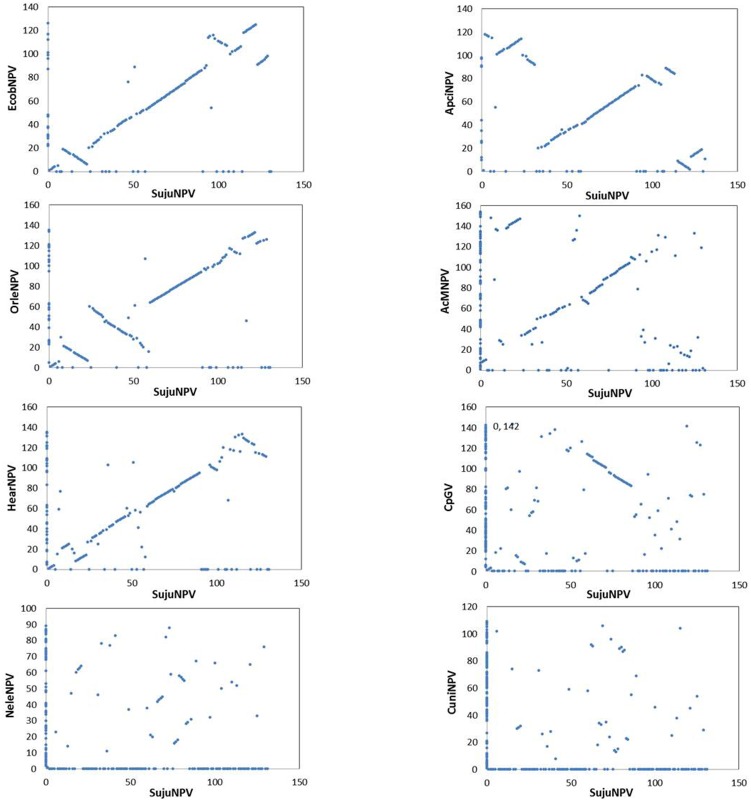
Gene-parity plot analysis. Gene-parity plots of SujuNPV against three close viruses (EcobNPV, ApciNPV and OrleNPV) and five representative baculoviruses (AcMNPV, HearNPV, CpGV, NeleNPV and CuniNPV).

Gene-parity plots of SujuNPV against three viruses in the same subclade and the five representative baculoviruses are shown in Fig. 3. The gene order between SujuNPV and ApciNPV, EcobNPV or OrleNPV revealed a high collinearity along the genomes, with some inversions and drifts. The plots of SujuNPV with representative lepidopteran baculoviruses (AcMNPV, HearNPV and CpGV) showed that SujuNPV is largely collinear with AcMNPV and HearNPV, less collinear with CpGV, but all contains a collinear region from Suju60 to Suju86, containing 20 core genes and five additional lepidopteran baculovirus conserved genes. This region has been suggested to exist in the ancestor of lepidopteran baculoviruses [27]. No obvious collinear region could be found between SujuNPV and NeleNPV or CuniNPV (Fig. 3).

### Homologous regions

Homologous regions (*hrs*) are common elements in many baculoviruses, with characteristically high A+T contents, tandem repeats and imperfect palindromes. *Hrs* vary in location within genomes, number of copies and nucleotide sequences between different baculoviruses. These regions are suggested to act as replication origins and transcription enhancers [28], [29].

**Figure 4:**
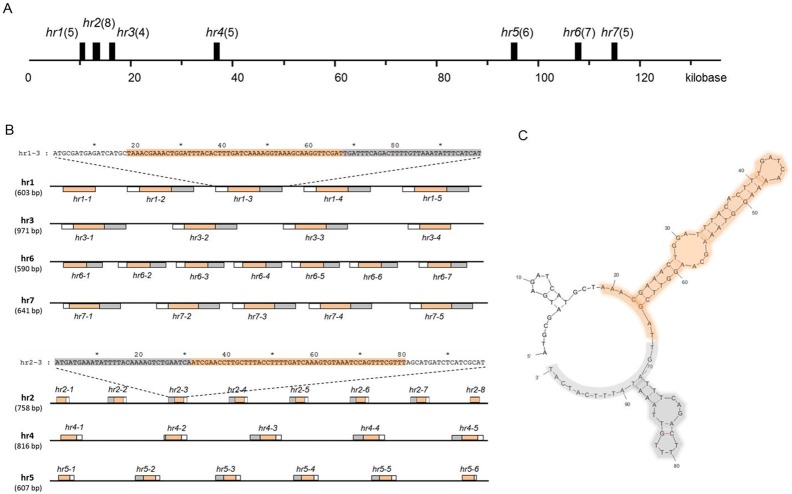
Analysis of SujuNPV *hr*s. **A.** The location and distribution of *hr*s in the SujuNPV genome. Black bars indicate *hr*s in the SujuNPV linear map. The number in brackets refers to the number of palindrome repeats in the homologous region. **B.** The arrangement of palindrome repeats in each homologous region. The rectangle above or below the black line indicates the orientation of repeats and number in the bracket represent the corresponding size of each *hr*. Different colors means the different fragments within the repeats, and orange indicates the core palindrome region, which is conserved in all the hr repeats. One sequence of each orientation was displayed at the top as a sample. **C.** The second structure of the *hr1–3* palindrome repeats. The background color is in line with the sequence displayed in Fig. 4B.

The SujuNPV genome contains seven homologous regions, covering 3.7% of the genome, as displayed in Fig. 4A. The length of the *hrs* ranges from 590 bp-971 bp, and each *hr* consists of four to eight palindromic repeats of 99 bp in length (Fig. 4A and 4B). Fig. 4B shows the arrangement of palindrome repeats in each homologous region. These palindromic repeats share at least 97.6% identity. The predicted secondary structure of the *hr1–3* revealed that it contains a core palindrome region, colored by orange in Fig. 4C, and it is highly conserved in all counterparts, with about 99.5% identity on average. While the other two loop were not such conservative, neither on the size nor sequence.

### DNA replication genes

Five core genes, five additional lepidopteran baculovirus conserved genes and eight other common genes involved in DNA replication were found in the SujuNPV genome (Table 2) [30]–[32]. Among these genes: *helicase* unwinds DNA [32]; *dna polymerase* is involved in DNA synthesis; late expression factor gene 3 (*lef-3)* and DNA binding protein gene (*dbp*) are involved in single-strand binding [33], [34]; *dna-ligase* in ligation and alkaline exonuclease (*alk-exo*) in rectification [35], [36], together with some other stimulators are required in the process of replication.

**Table 2:** Classification of gene function.

	Core genes	Lepidoptera baculovirus conserved genes	Common genes
**Replication**	alk-exo(Suju125), dna polymerase(Suju63), helicase(Suju79), lef-1(Suju121), lef-2(Suju110)	dbp-1(Suju30), ie-1(Suju23), lef11(Suju28), lef-3(Suju61), me53(Suju16)	dbp-2(Suju13), dnaphotolyase(Suju131), dna-ligase(Suju50), endonuclease(Suju92), helicase-2(Suju57), ie-0(Suju17), nrk1(Suju94), parg(Suju98),
**Transcription**	lef-4(Suju74), lef-5(Suju82), lef8(Suju33), lef-9(Suju49), P47(Suju31), vlf-1(Suju66)	39k(Suju27), lef-6(Suju12), pk-1(Suju3)	lef12(Suju32)
**Structure**	38k(Suju81), ac53(Suju38), ac78(Suju67), ac81(Suju69), desmoplakin(Suju62), gp41(Suju68), odv-e18(Suju19), odv-e25(Suju78), odv-ec27(Suju20), odv-ec43(Suju89), p18(Suju77), p33(Suju76), p40(Suju84), p48/p45(Suju86), p49(Suju18), p6.9(Suju83), vp1054(Suju41), vp39(Suju73), vp91(Suju71)	F(Suju115), fp/25k(Suju48), orf1629(Suju2), p12(Suju85), p24(Suju108), polyhedrin(Suju1), tlp-20(Suju70)	calyx/pep(Suju104), cg30-1(Suju72), cg30-2(Suju8), p10(Suju9), pkip(Suju111), vp80(Suju87)
**Auxiliary**		fgf(Suju127)	bro-1(Suju7), bro-2(Suju47), bro-3(Suju51), bro-4(Suju130), chitinase(Suju54), egt(Suju119), gp37(Suju52), iap-1(Suju36), iap-2(Suju59), iap-3(Suju96), sod(Suju102), ubiquitin(Suju26), v-cath(Suju55)
***Pifs***	p74(Suju15), pif-1(Suju129), pif-2(Suju113), pif-3(Suju100), pif-4(Suju80), pif-5(Suju6), pif-6(Suju60)		
**Unknown**		38.7k(Suju122), ac106(Suju97), ac110(Suju88),ac75(Suju64), ac76(Suju65), chtb(Suju21), ep23(Suju22), bv-e31(Suju29)	ac108(Suju90), ac111(Suju114), ac112(Suju93), ac117(Suju103), ac150(Suju58), ac17(Suju117), ac19(Suju123), ac29(Suju11), ac34(Suju24), ac52(Suju37), ac55(Suju42), ac56(Suju43), ac57(Suju44), chaB(Suju45), chaB(Suju46), djbp(Suju35), hoar(Suju4), p26-1 (Suju10), p26-2(Suju56), p43(Suju95), Suju101, Suju105, Suju107, Suju109, Suju112, Suju116, Suju118, Suju120, Suju124, Suju126, Suju128, Suju34, Suju39, Suju40, Suju75, Suju91, Suju99

Functional classification of the genes in the SujuNPV genome columns indicate classification by function and rows represent conservatism. Genes in the SujuNPV genome were arranged according to their functions and conservatism in alphabetical order.

Functional classification of the genes in the SujuNPV genome; columns indicate classification by function and rows represent conservatism. Genes in the SujuNPV genome were arranged according to their functions and conservatism in alphabetical order.Some common genes involved in baculovirus replication were not present in SujuNPV. For example, *lef-7* which has been shown to be a replication enhancer in baculoviruses [37], was absent from SujuNPV. SujuNPV also lacked certain genes associated with nucleotide biosynthesis, such as the ribonucleotide reductase subunits (*rr1*, *rr2*) and *dUTPase*, which are involved in dTTP biosynthesis [38].

**Figure 5:**
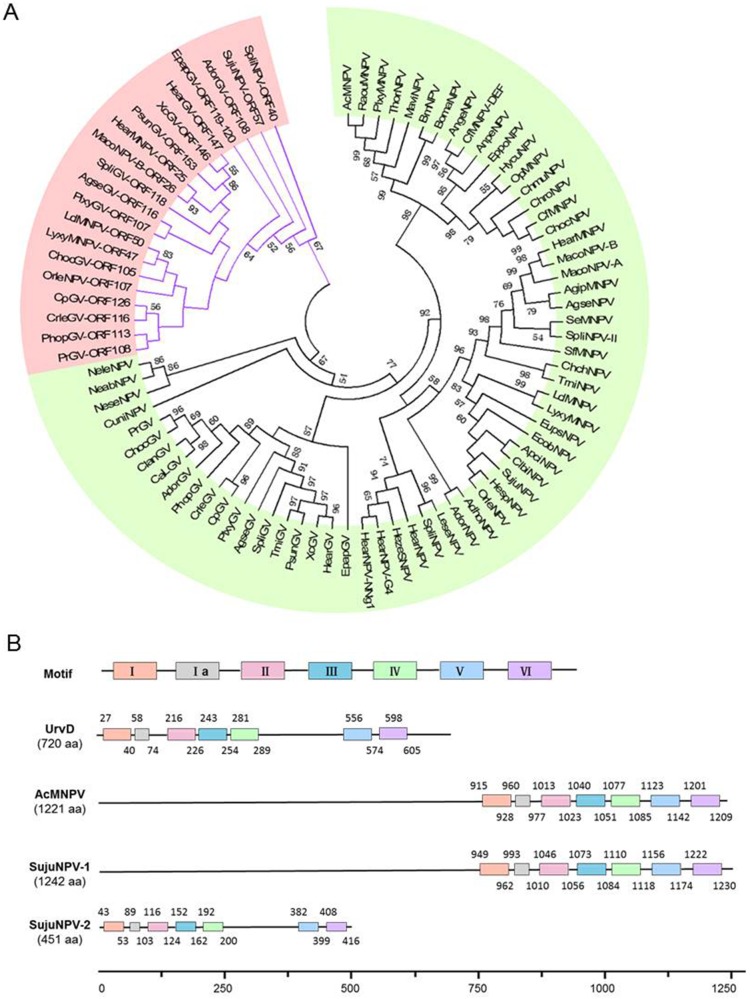
Analysis of the duplicated gene helicase and its conservative motifs. **A.** The tree was reconstructed based on protein sequences by MEGA5. The second copy was colored by purple branches and pink background and the number on the branch indicates a bootstrap value of 1000 randoms. **B.** Conservative motifs of *E.coli* UrvD, AcMNPV, SujuNPV Helicase (SujuNPV-1) and SujuNPV Helicase-2 (SujuNPV-2) were displayed. The blank line indicates the relevant protein with length in the bracket. The colored boxes on the line indicate motifs I-IV and the numbers above and below the box mean the start and end position of each motif in the protein, respectively.

Amongst the DNA replication genes, there are two copies of *helicase* and *dbp* in the SujuNPV genome. A full length *helicase* (Suju79, 1242aa) is a core gene found in all sequenced baculoviruses, whilst a second copy of truncated *helicase* (*helicase-2)* (Suju57, 451aa) is present in only six alphabaculoviruses (HearMNPV, LdMNPV, LyxyMNPV, MacoNPV-B, OrleNPV and SpliNPV) and 13 GVs (all sequenced GVs except for ClanGV and CaLGV) [39]. The phylogenetic tree of *helicase* homologies showed that they can be clearly divided into two groups (Fig. 5A). It is very likely that they were acquired from different sources during evolution. The research of AcMNPV *helicase* reveals that it belongs to Superfamily 1 helicase, which contain 7 conserved motifs [40], [41]. Motifs I and II are two NPT-binding motifs, together with another four motifs to fulfill the function of helicase [42], [43]. The alignment of the conserved motifs with AcMNPV and *E.coli* UrvD (representative of Superfamily 1 *helicase*) reveals that they share the same motifs (Fig. 5B) and that *helicase-2* is seemingly more conservative. It appears that the two copies have a common ancestor, but understanding how they evolved and came to balance their specialization and cooperation within one genome requires further research.

**Figure 6:**
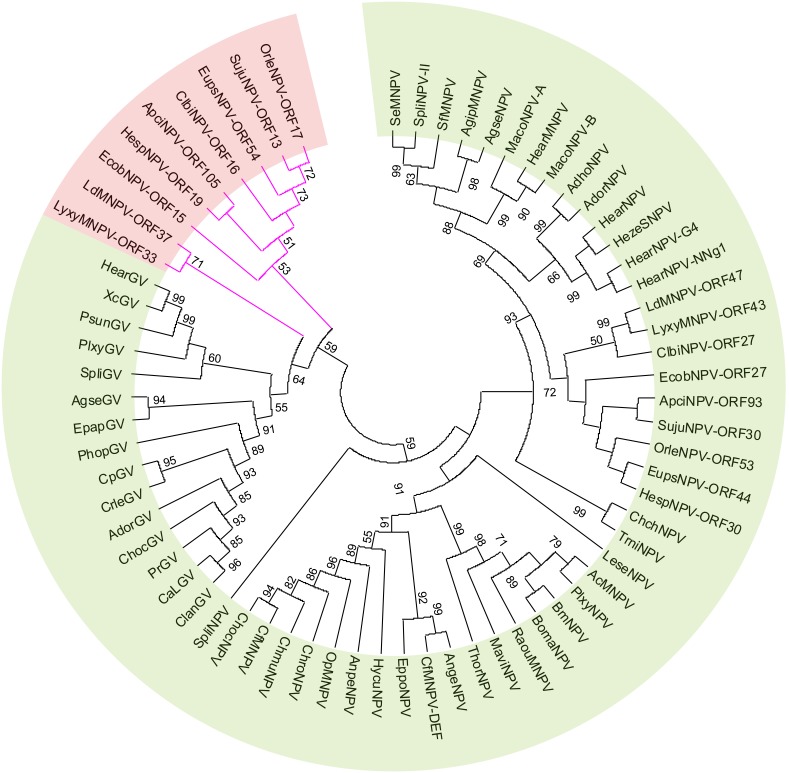
Analysis of the duplicated gene *dbp*. The tree was reconstructed based on protein sequences by MEGA5. The second copy was colored by purple branches and pink background and the number on the branch indicates a bootstrap value of 1000 randoms.

SujuNPV is the ninth baculovirus identified to have double copies of *dbp*; the other eight are ApciNPV, ClbiNPV, EcobNPV, EupsNPV, HespNPV, LdMNPV, LyxyMNPV and OrleNPV. Interestingly all these viruses belong to the same subclade (Fig. 2). *Dbp* is a conserved gene in lepidopteran baculoviruses. Phylogenetic analysis indicated that the *dbp* duplicates of these nine baculoviruses may have evolved separately to the conserved *dbp* in alphabaculoviruses (Fig. 6). We propose to name the alphabaculovirus-conserved *dbp* gene as *dbp*-1, and the second copy as *dbp*-2. *Dbp-2* appears to be more close to the *dbp* of betabaculovirus. In SujuNPV, *dbp-1* (Suju30) *and dbp-2* (Suju13) encode 309 aa and 310 aa proteins respectively, with 25% aa identity. Although the significance of SujuNPV and other bacuoviruses carrying two copies of *dbp* is unclear, it clearly marks out the subclade of these nine group II alhpabaculoviruses.

### Transcriptional genes

In a baculovirus life cycle, the genes are transcribed in cascades by different polymerase. Early stage genes are transcribed by host RNA polymerase II, while genes expressed during the late period of the life cycle are transcribed by the virus-encoded RNA polymerase, comprising four core gene transcripts: LEF-4, LEF-8, LEF-9, P47 [44]. Two other core genes are involved in late phase transcription: *lef-5* and very late factor (*vlf-1*), acting as an initiation factor [45] and a regulatory factor participating in the hyper-expression of very late genes [46], respectively. These core genes, in addition to genes such as *39k, lef-6*, *lef-10* and *lef-12*, are required for late transcription [47]. All of these genes appear in SujuNPV, except for *lef-10* (Table 2) and among all the other alphabaculoviruses this gene was only absent from ClbiNPV and OrleNPV.

### Structural genes

**Figure 7:**
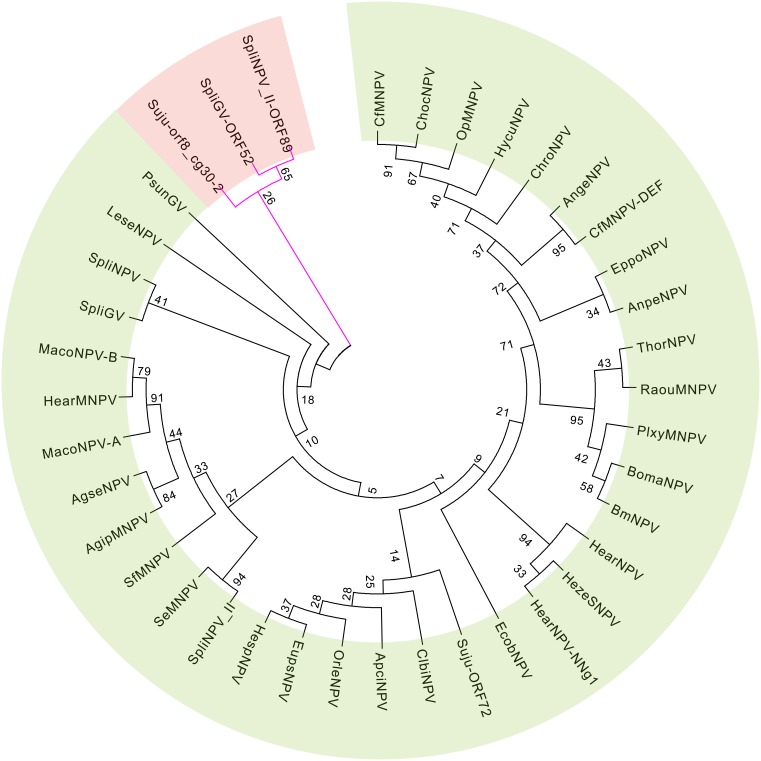
Analysis of the duplicated gene *cg30*. The tree was reconstructed based on protein sequences by MEGA5. The second copy was colored by purple branches and pink background and the number on the branch indicates a bootstrap value of 1000 randoms.

Nineteen core genes and seven additional lepidopteran-conserved genes related to structure were found in the SujuNPV genome (Table 2) [48]–[50]. In addition, six other common genes were also identified in the SujuNPV genome (Table 2). *Cg30* is duplicated in SujuNPV: *cg30-1* (Suju72, 315 aa) and *cg30-2* (Suju8, 272 aa). Among all the baculoviruses sequenced, two copies of *cg30* are only present in SpliNPV-(SpliNPV82 and SpliNPV89) and in SpliGV (SpliGV52 and SpliGV124). *Cg30-1* of SujuNPV has many homologies with other baculoviruses, while *cg30-2* groups with SpliNPV89 and SpliGV52 at the outmost of the phylogenetic tree (Fig. 7), sharing an aa identity of 15% and 14%, respectively.

### *Per os* infectivity factors

So far seven genes have been identified as *per os* infectivity factors (PIFs), including *p74*, *pif1*, *pif2*, *pif3*, *pif4* (*odv-e28*), *pif5* (*odv-e56*) and *pif6,* which are essential for the oral infection of insect larvae [51]–[54]. PIF-1, PIF-2 and PIF-3 in association with P74 form a conserved complex on the surface of ODV and were proposed to perform an essential function in the early stages of virus infection [51]. PIF-4 is an envelope-associated protein found in both ODV and BV [52], whereas, PIF-5 and PIF6 have been recently demonstrated to be PIF members [53], [54]. All seven of their genes are conserved within the SujuNPV genome and share 44%–61.2% identity with their homologues in group II representative baculovirus HearNPV.

### Auxiliary genes

Auxiliary genes are those not essential for replication, transcription or structures, but provide the virus with the stronger adaptive ability [55], such as affecting the host’s cellular metabolism for successful infection or by promoting the progeny yields of the virus. Examples are fibroblast growth factor (*fgf*) and *gp37*, which are proposed to help to spread virions from the primary infection site [56], [57], *egt*, which promotes viral progeny by delaying larval molting [58], and *cathepsin* and *chitinase*, which aid the horizontal spread of viruses [59]. Superoxide dismutase (*sod*) has been suggested to migrate the effects of free radicals in infected hemocytes [60] and *ubiquitin* is proposed to stabilize viral proteins against being degraded by hosts [61]. Among these auxiliary genes, no core gene has been found and only *fgf* is a lepidopteran-conserved gene. SujuNPV was found to contain all the genes above (Table 2).

Anti-apoptosis genes are those encoded by viruses in order to resist the programmed death of infected cells, hence ensuring a successful infection [62]. SujuNPV possesses two types of anti-apoptotic genes: *p49* (Suju18) and three copies of inhibitor of apoptosis gene (*iap*s): *iap-1* (Suju36), *iap-2* (Suju59) and *iap-3* (Suju96). Among the three *iap*s, *iap-2* and *iap-3* have a C_3_HC_4_ motif at the C terminal, with DNA-binding properties [63].

Baculovirus repeated ORFs (*bros*) are repetitive genes, which are widespread in baculoviruses and some other insect virus DNA [64]. Research of BmNPV showed that *bros* contained DNA-binding activity that could influence host DNA replication and transcription [65]. Four *bro* genes were identified in SujuNPV, and named *bro-1* to *bro-4*, based upon their order of appearance in the genome (Fig. 1). SujuNPV *bro-3* had an aa similarity to its homologues in AcMNPV, OpMNPV, LdMNPV and HearNPV-G4, with 36%, 38.6%, 35.2% and 13.3% sequence identity, respectively. The other three *bro*s only shared a C-terminal region with Ld-bro-m, Ld-bro-p and Ld-bro-n.

### Unknown genes

**Figure 8:**
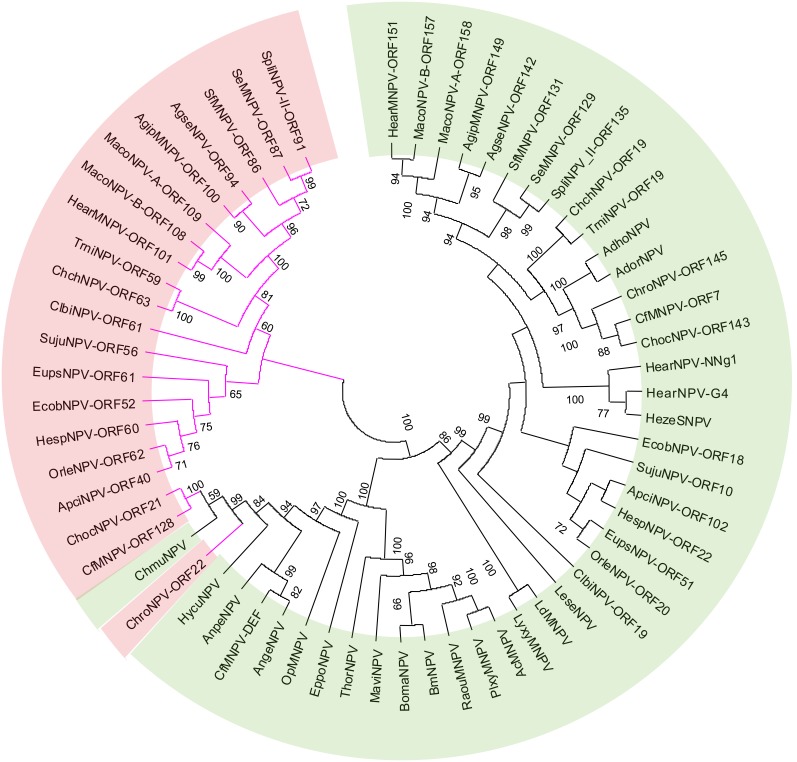
Analysis of the duplicated gene *p26*. The tree was reconstructed based on protein sequences by MEGA5. The second copy was colored by purple branches and pink background and the number on the branch indicates a bootstrap value of 1000 randoms.

SujuNPV contained an additional eight *lepidoptera*-conserved genes and 37 common genes with unknown functions (Table 2). *P26* is an alphabaculovirus-specific gene. Among the 42 alphabaculoviruses previously sequenced, 19 contained a second copy of *p26* and 16 of these belonged to group II. SujuNPV also contains two copies of *p26*, Suju10 (*p26-1*, 285 aa) and Suju56 (*p26-2*, 239 aa), which share 13.8% similarity. We name the one conserved in alphabaculoviruses as *p26-1*, and the second copy as *p26-2*. Phylogenetic analysis of *p26* showed that the second copies of *p26* could be classified into a unique subclade (colored pink in Fig. 8), with the exception of three group I baculoviruses (CfMNPV, ChocNPV and ChroNPV). Interestingly, the group II baculoviruses, except for LeseNPV, all specifically contain a conserved gene cluster that is *p10*, *p26*, *ac29*, *lef-6* and *dbp* (*dbp-2* in the 9 *dbp*-duplicated baculoviruses) in order. Although the significance of this gene cluster is unknown, it can provide us with more information for evolutionary analysis.

### Unique genes

Five genes are unique to the SujuNPV genome, including Suju5 (353 aa), Suju14 (83 aa), Suju25 (192 aa), Suju53 (220 aa) and Suju106 (174 aa) which were not included in Table 2. Suju5 has a similar location and length to the ORF5 of Buzura suppressaria SNPV (BusuNPV) with14.8% aa identity, indicating they may have similar function [27]. Suju25 has an early promoter and a BLAST search showed it to have a slight similarity to the ATP-binding protein of *Lysinibacillus sphaericus* C3–41 with an E-value of 0.89. No homologues were found in GenBank for the other three ORFs, whether these are functional ORFs of SujuNPV requires further experimentation.

## Conclusion

Our analyses revealed that SujuNPV is a novel baculovirus within a unique subclade of group II alphabaculovirues, the members of which all contain a second copy of *dbp*. The SujuNPV genome contains seven *hrs* and five unique ORFs, as well as several genes with two or more copies. The presence of duplicated genes in this virus raises the question on the mechanisms of its acquisition (duplication of virus genes or independent horizontal transfer) and maintain, which needs further researches. These findings will facilitate future applications of SujuNPV to pest control and provide new data for the elucidation of the evolutionary pathways of baculoviruses.
